# When Distance Matters: The Quadro-iliac Plane Block in Complex Multilevel Posterior Spinal Fusion Surgery

**DOI:** 10.4274/TJAR.2026.252367

**Published:** 2026-06-26

**Authors:** Hande Gürbüz, Polen Nurdan Şen, Çağdaş Baytar

**Affiliations:** 1 University of Health Sciences Türkiye, Bursa City Hospital, Clinic of Anaesthesiology and Reanimation, Bursa, Türkiye; 2 Zonguldak Bülent Ecevit University, Department of Anaesthesiology and Reanimation, Zonguldak, Türkiye

Dear Editor,

Complex multilevel spinal surgeries are associated with severe postoperative pain due to extensive paraspinal muscle dissection, prolonged operative time, and multilevel instrumentation. Consistent with our prior institutional experience and the literature, patients undergoing multilevel spinal fusion without regional analgesia demonstrate an increased need for rescue opioid analgesia.^1^ Although several regional techniques have been proposed, effective options that do not interfere with the surgical field remain limited. Thoracolumbar interfascial plane and erector spinae plane (ESP) blocks are commonly used; however, their efficacy may be compromised in multilevel procedures where surgical trauma, edema, and instrumentation disrupt local anatomy and impair local anaesthetic spread.^1^

In this context, the quadro-iliac plane (QIP) block may overcome some of these limitations.^2, 3, 4^ It targets the posterior aspect of the quadratus lumborum muscle at its iliac crest attachment, enabling, with a single injection, the spread of local anaesthetic into the interfascial planes between the erector spinae and quadratus lumborum and between the quadratus lumborum and psoas muscles, thereby providing a plausible basis for lumbosacral paraspinal analgesia. While QIP block has shown efficacy in single-level lumbar discectomy and may be non-inferior to ESP block,^5^ evidence in multilevel spinal stabilization remains limited to a few small case series involving patients undergoing three-level spinal instrumentation.^3, 6^

Based on this rationale, QIP block was applied in four consecutive patients undergoing multilevel posterior spinal fusion and decompression via laminectomy at a single institution as part of routine clinical practice, as previously described (Figure 1).^2^ Following informed consent, a bilateral ultrasound-guided QIP block was performed postoperatively using 20 mL of 0.25% bupivacaine per side. Patients were monitored in the recovery unit for approximately four hours. Postoperative analgesia was standardized as an alternating regimen of dexketoprofen and paracetamol every 12 hours; rescue opioids were administered if pain scores exceeded 3.

The cohort included three female patients and one male patient (aged 61-73 years), all undergoing four-level stabilization (T11-L2, L1-L4, and two at L2-L5). All patients received scheduled non-opioid analgesics, and none required rescue opioids. Pain scores remained low at rest [numeric rating scale (NRS) 1-2] and did not exceed NRS 3 with movement. One patient with T11-L2 instrumentation reported mild pain at the cranial extent of the incision during the first postoperative hour, suggesting limited cranial coverage; however, the discomfort remained tolerable. All patients were able to reposition comfortably, showed no motor blockade, and were mobilized on the first postoperative day.

In practice, a QIP block applied at a site distant from the operative area was readily accepted by the surgical team and addressed concerns regarding blocks performed near fresh posterior incisions. Moreover, observations from our four cases suggest a potential opioid-sparing effect in this surgical population, and this effect was well received by both the surgical and  anaesthesiology  teams and by the patients.

In the cadaveric study by Tulgar et al.,^2^ injection of 40 mL of dye per side demonstrated spread extending from the iliac crest to the 12^th^ rib, involving the transversalis fascia, adjacent retroperitoneal fat, and mid-level lumbar plexus structures.^2^ This distribution may explain the limited cranial coverage observed in the T11-L2 case. Furthermore, despite the use of a lower volume of local anaesthetic, effective analgesic coverage from L1 to L5 was achieved in our cases. Although cadaveric findings suggest a theoretical risk of motor blockade due to lumbar plexus involvement, no motor blockade was observed in our patients, possibly because of the lower volume and concentration used. However, this risk should be considered when reliable postoperative neurological assessment is required.

Finally, patients undergoing multilevel spinal fusion often have chronic back pain, which may alter thoracolumbar fascia micro- and macro-structure and function, thereby influence the spread, diffusion, and absorption of local anaesthetics and lead to variability in block efficacy;^7^ thus, beyond cadaveric findings these factors should be taken into account in clinical practice.

Evidence for QIP block in multilevel spinal stabilization remains limited to a few case reports, including our four cases. Thus, these findings should be considered hypothesis-generating rather than generalizable, and further prospective studies are needed to clarify its role.

## Figures and Tables

**Figure 1:**
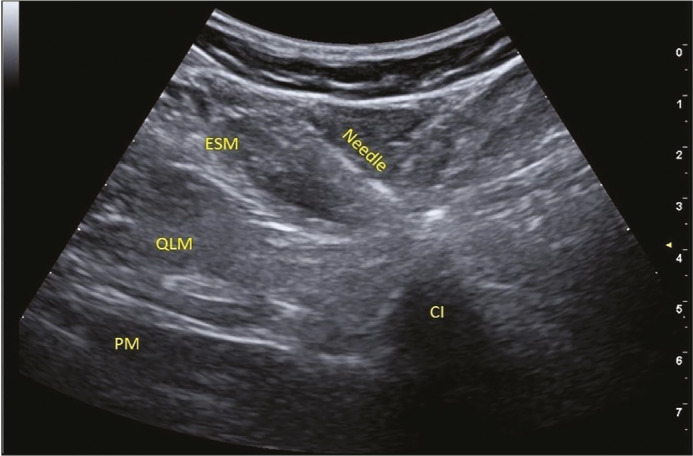
Quadro-iliac plane block-ultrasound view. CI, crista iliaca; ESM, erector spinae muscle; PM, psoas muscle; QLM, quadratus lumborum muscle.

## References

[1] Kaciroglu A, Ekinci M, Gurbuz H (2024). Surgical vs ultrasound-guided lumbar erector spinae plane block for pain management following lumbar spinal fusion surgery.. Eur Spine J.

[2] Tulgar S, Ciftci B, Ahiskalioglu A (2024). Ultrasound guided quadro-iliac plane block: another novel fascial plane block.. Pain Med.

[3] Ciftci B, Cetinkal A, Alver S, Ahiskalioglu A (2025). Quadro-iliac plane block for lumbar multi-level instrumentation surgery: far away from the surgical area.. Minerva Anestesiol.

[4] Bilal B, Çiftçi B, Ahiskalioğlu A, Özdemir M, Çalişir F, Tulgar S (2025). Serratus posterior superior intercostal plane block + quadroiliac plane block for scoliosis surgery: two novel blocks away from the surgical field.. Minerva Anestesiol.

[5] Turan Eİ, Bıyıkoğlu BO, Özen V (2026). Comparison of quadro-iliac plane block and erector spinae plane block for postoperative analgesia management after single level lumbar discectomy surgery: a randomized, double-blind, controlled, prospective, multicenter study.. J Anesth.

[6] Turan Eİ, Şahin AS (2025). Quadro-iliac plane block (QIPB) in lumbar stabilisation surgeries: a case series.. Indian J Anaesth.

[7] De Cassai A, Marrone F, Sun Y (2025). Histology of the fascial planes: a systematic review of the microstructural foundations of regional anesthesia.. J Anesth Analg Crit Care.

